# The effect of weight reduction on the diabetes foot: A scoping review and clinical implications

**DOI:** 10.1016/j.obpill.2024.100152

**Published:** 2024-12-06

**Authors:** Janine E. Roll Ahmed, John Veto, Derek Santos

**Affiliations:** a Queen Margaret University (QMU), Queen Margaret University Drive, Musselburgh, EH216UU, Scotland, UK; b University of Stirling, Podiatry Department, UK; c QMU, Podiatry Department, Queen Margaret University Drive, Musselburgh, EH216UU, UK

**Keywords:** Diabetes, Weight reduction, Foot

## Abstract

**Background:**

This scoping review aims to explore the literature available on rapid weight reduction (either via diet/exercise or bariatric surgery) on the diabetes foot.

**Methods:**

A search strategy was developed using Population, Intervention, Comparison, Outcome (PICO). A search using all identified keywords and index terms was performed on Cumulated Index in Nursing and Allied Health Literature (CINAHL), Medical Literature Analysis and Retrieval System Online (MEDLINE), Excerpta Medica Database (EMBASE), PubMed, International Clinical Trials Registry Platform (ICTRP) and Web of Science (WoS) databases as well as Proquest, and Cochrane systematic reviews. A search of grey literature was also performed on Google Scholar and Internet explorer. No restrictions were placed on the date of publication.

**Results:**

There were few studies that explored the effects of weight reduction on the diabetes foot. Only 2 articles met the scoping review's inclusion criteria. These were a case study where the intervention was bariatric surgery and a case series study where the interventions were bariatric surgery or diet and exercise.

**Conclusion:**

This scoping review has highlighted the lack of research in this area, raising opportunities for further research that focus on the effects of rapid weight reduction (either following bariatric surgery or weight reduction and diet) on the diabetes foot. Patients with diabetes mellitus should receive individualised pre and post weight reduction care to their feet, either by a podiatrist or multidisciplinary care team.

## Graphical abstract

**Figure:**
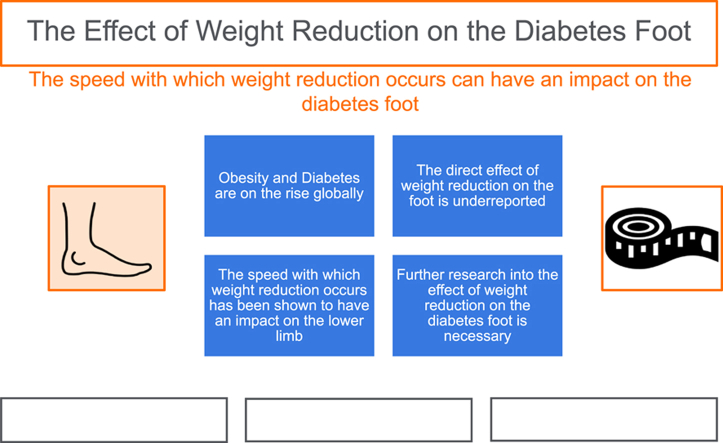

## 1 Introduction

The global prevalence of diabetes mellitus has increased dramatically over the past several decades. Approximately 537 million adults are living with diagnosed diabetes mellitus worldwide, with the total number of people with the disease projected to rise to 643 million by 2030, and 783 million by 2045 [1]. Characterised by a chronic state of hyperglycemia, the presence of glucosuria and alterations in systemic insulin regulation [2] the metabolic disease can cause systemic complications such as retinopathy, cardiovascular disease [3], peripheral neuropathy, nephropathy and kidney failure. It can also cause extensive destruction of the lower limb [4], leading to amputation and other insidious long-term outcomes being a possibility that may contribute to a person living with a lifelong disability [5].

With regard to the current literature available, it is feasible to say that in patients without diabetes, weight reduction by whatever means can potentially increase strength, balance, gait speed, as well as change the plantar pressures within the foot. Despite this, weight reduction in patients without diabetes also has the potential to increase the risk of adverse outcomes post weight reduction. A study by Weyns et al. [6] demonstrated that in patients without diabetes, significant and rapid weight reduction was correlated with a higher risk of developing foot drop. In patients with diabetes mellitus, there is no consensus as to whether existing foot disease improves with substantial weight reduction [7]. These unresolved effects of weight reduction even in the healthy patient, allude to the necessity for further research on the topic.

As a metabolic disease, diabetes mellitus is progressive in nature and its pathophysiological changes often start some years before a patient is diagnosed [8]. There is evidence to indicate that in patients whose diabetes mellitus condition improves, the risk of foot disease remains [7]. This will occur even if a patient's diabetes mellitus has gone into remission as the systemic destruction has already taken place. A myriad of problems can occur in patients with diabetes mellitus who experience weight reduction without pre-emptive and ongoing foot care, particularly those with pre-existing neuropathy and macro/micro vascular disease [9].

For the patient with overweight or obesity with diabetes mellitus, an elevated risk of osteoarthritis (OA) is ever present both as a consequence of diabetes mellitus as a systemic disease, and the increased force exerted through weight bearing joints due to the patient being overweight or obese [10]. For patients with diabetes mellitus living with OA, losing weight is of prominent importance in slowing the progression of the disease [11]. OA frequency exists amongst patients with diabetes mellitus and there is an association between both diseases. Further research on the effect of weight reduction will identify if OA symptoms in the foot of the patient with diabetes mellitus can be improved, or progression of the disease delayed if weight reduction is the resulting outcome.

In the finite literature on the topic, it has been established that bariatric surgery as a form of weight reduction can lead to a remission of diabetes mellitus and can actually assist to cease the progression of peripheral neuropathy [12]. However, there is insufficient data to conclude that it can improve or reverse the condition. To discern how these outcomes collectively effect the patients’ feet, a scoping review of the limited literature on the subject needs to be undertaken for further clarity.

## 2 Method

### 2.1 Justification for a scoping review

At present very little in the way of directives and comprehensive literature exist for the patient with diabetes mellitus (and for the practitioner or multidisciplinary team treating them) when referring to weight reduction and how it effects the feet. This scoping review is necessitated by an inherent lack of literature on the topic and a requirement that the subject matter relating to it to be reviewed.

A scoping review aims to map the existing literature in a field of interest [13] and can be particularly useful when a topic or subject matter has not yet been extensively reviewed or is of a complex nature [14]. Scoping reviews are commonly undertaken to examine the extent, range, and nature of research activity in a topic area [15]. This scoping review will be undertaken to assist in determining the value and potential scope of undertaking a full systematic review [16]; summarize and disseminate research findings; and identify research gaps in the existing literature [13].

In 2021, Diabetes Feet Australia [17] produced a set of evidence-based guidelines, outlining how diabetes foot related disease should be approached. These guidelines whilst dealing with prevention, wound classification, infection and treatment options, do not refer to an approach to footcare pre and post weight reduction that is comprehensive and preventative for the patient with diabetes mellitus.

### 2.2 Scoping review question

In particular, this scoping review will explore the current literature relating to the patient with overweight or obesity living with diabetes mellitus. In this way, it is hoped that the outcome of weight reduction and its subsequent effect on a patient's feet can be analysed. In an extension of that, does weight reduction resulting from bariatric surgery provide a different outcome for the patient with diabetes' feet? Where gaps in the literature are evident, recommendations for pre-emptive and long-term care and avenues for further research on the specific topic will be given.

### 2.3 Literature search process

The literature search process for scoping the research question was guided by the Population, Intervention, Comparison, and Outcome (PICO) framework. The format was used to define the research question for the review as follows: What is the effect of weight reduction on the diabetes foot? Where the [population] are patients with overweight or obesity with diabetes mellitus, the [intervention] is the weight reduction either by diet and/or exercise or bariatric surgery, the [outcome] is the effect on the feet, and the comparison is no comparative intervention.

### 2.4 Search strategy

An electronic search was undertaken on Queen Margaret University library databases on 16 August 2022. The databases incorporate Cumulated Index in Nursing and Allied Health Literature (CINAHL), Medical Literature Analysis and Retrieval System Online (MEDLINE), Excerpta Medica Database (EMBASE), Pubmed, International Clinical Trials Registry Platform (ICTRP), ProQuest and Web of Science (WoS) and Cochrane systematic reviews. The databases were comprehensively searched from their inception dates up to the present day and have been selected for their appropriateness to the topic and relatedness in identifying medical and allied health intervention studies both current and retrospective. A structured search of the literature on the topic was limited to English language articles as the interpretation of non-English language articles incorporates the use of resources that is not within the scope of this review.

Each PICO element was entered into the database search engine separately using Boolean operator ‘OR’ to combine each listed synonym [18]. The asterisk (∗) was also used for truncation where necessary. The resulting searches were then combined with other search terms using the Boolean operator ‘AND’.

### 2.5 CINAHL, MEDLINE, EMBASE, PUBMED, ICTRP and WEB of SCIENCE search strategy

The following search strategy was carried out: (1) diabet∗; (2) obes∗; (3) overweight; (4) bariatric; (5) 1 or 2 or 3 or 4; (6) weight reduction; (7) fat loss; (8) diet∗; (9) exercise; (10) gastric sleeve; (11) gastric bypass; (12) weight reduction surgery; (13) 6 or 7 or 8 or 9 or 10 or 11 or 12; (14) foot; (15) feet; (16) 14 or 15. These were then combined using AND, that is (5) AND (13) and (16).*2.6 Proquest search strategy*.

The following search terms were used: Weight reduction AND diabetes AND foot AND (obesity AND overweight).

### 2.6 Cochrane systematic reviews

The following search was undertaken: Diabetes AND weight reduction AND foot. This search resulted in no publications being found reporting on the effects of weight reduction on the diabetes foot.

### 2.7 Grey literature

Searches undertaken on Google scholar and Internet Explorer were undertaken using the search terms: Diabet∗, weight reduction, and feet.

### 2.8 Review methods

After undertaking the database searches, one reviewer JA independently imported the titles and abstracts as RIS files into the Rayyan application. Potentially eligible studies were retrieved and independently and blindly screened by title and abstract by JA and DS after duplicates were removed. The authorship of articles and results were made fully disclosed at the time of screening. The inclusion of articles was agreed upon by JA and DS unblinded and any differences and reasons for exclusion were discussed after reviewing the full text articles. The consultation of a third reviewer (JV) was not required, as both JA and DS agreed on the articles for inclusion to the scoping review.

JA and DS independently extracted data from included studies using a standardised pilot tested form. Inconsistencies in the data extracted were discussed between JA and DS. As there are only two articles that are of clear significance to be included into this scoping review, both articles were considered based on the population/sample, study design, aim or objective, intervention, outcome measures, results, and limitations. Both articles were found to have a high risk of bias.

### 2.9 Inclusion criteria

For the purposes of this scoping review, the population are patients with overweight or obesity with diabetes mellitus, the intervention is the weight reduction either by diet and/or exercise or bariatric surgery, the outcome is the effect of weight reduction on the feet, and the comparison is no comparable intervention.

### 2.10 Exclusion criteria

Articles or abstracts not written in the English language.

### 2.11 Results from search strategy

**Fig. 1:**
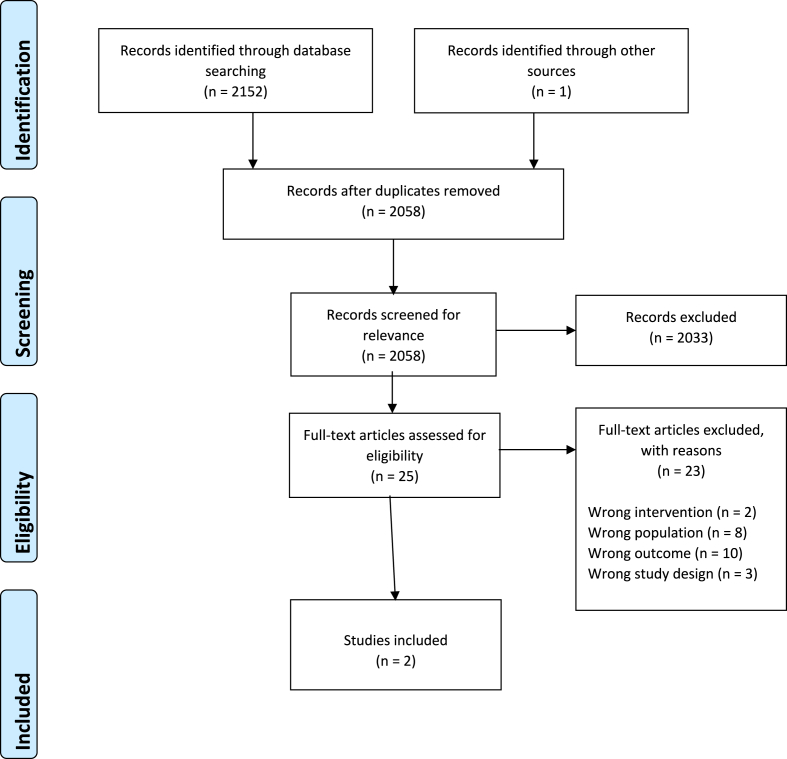
PRISMA flow diagram.

Following the electronic searches undertaken on the databases described, 2152 papers were identified. One paper was obtained via the searching of grey literature. After duplicates were removed, 2058 articles were screened for relevance to the topic [19]. Both JA and DS agreed that 2033 articles did not fulfil the inclusion criteria, and 25 articles remained to be assessed for eligibility. After consideration, 23 articles were excluded for having either the wrong intervention, wrong population, wrong outcome, or wrong study design. Please refer to the Prisma Flow diagram (Fig. 1) below for explanation.

### 2.12 Methodological bias

The risk of bias of the two studies included in this scoping review has been assessed by JA and DS using a checklist for the assessment of the methodological quality of randomised and non-randomised studies of health care intervention. As reported in Data Extraction Table 1, both studies included in this scoping review have been classified as being at a high risk of bias. This is due to the number of participants in each case study, the short duration of the studies, the lack of standardisation of inclusion and exclusion criteria and data collection periods. It is also important to comment that both studies included in this scoping review had the same authors.

**Table 1:** Data extraction table of studies.

(Author) Study	Population/Sample	Study Design	Aim/Objectives	Intervention	Outcome Measures	Results	Limitations
(Murchison et al., 2014) *The Development of a Charcot Foot after Significant Weight reduction in People with Diabetes: Three Cautionary Tales*	Patients with overweight/or obesity with type 2 diabetes mellitus.	Case studies	To report on 3 cases with type 2 diabetes mellitus that developed Charcot foot following weight reduction.	Weight reduction via the implementation of diet and exercise or bariatric surgery.	Reporting of foot complications following weight reduction in individuals with type 2 diabetes mellitus.	After weight reduction 3 individuals with type 2 diabetes mellitus developed a Charcot's neuroarthropathy, despite their diabetes improving significantly.	This study only involves three cases. A greater sample size is required for more generalisable results. Also, the period of time individuals developed the Charcot foot following weight reduction varied. In addition, some developed other complications post- surgery (e.g., osteomyelitis, ulcers, etc.) and not all suffered from neuropathy. A study is required to standardise inclusion/exclusion criteria as well as data collection periods. High risk of bias.
(Gooday et al., 2014) *Complex relationships requiring long-term follow-up: Obesity, bariatric surgery-induced diabetic remission, and the diabetes foot*	Patients with obesity with type 2 diabetes mellitus.	Case study	To report on one case with type 2 diabetes mellitus that developed Charcot foot following weight reduction.	Weight reduction via the implementation of bariatric surgery.	Reporting of foot complications following weight reduction in an individual with type 2 diabetes mellitus.	Post-surgery, the patient's diabetes went into remission, and she was more physically active. She subsequently developed Charcot's neuroarthropathy.	This study only involves one case. A greater sample size is required for more generalisable results. There were also other comorbidities that could have contributed (e.g., neuropathy) to the development of the Charcot foot. High risk of bias.

## 3 Discussion

The correlation of diabetes mellitus in the patient with overweight or obesity who has undergone weight reduction either through diet and/or exercise or bariatric surgery was first demonstrated by Gooday et al. [7]. In this single case study, bariatric induced diabetic remission and the effect of the diabetes foot was discussed. The patient, a type 2 diabetic (diagnosed in 1989) with prior foot pathologies underwent bariatric surgery and subsequently lost 46 kg. Despite significant improvements in the patient's diabetes control and subsequent improvement in mortality risk, neuropathy and foot deformity remained following bariatric surgery and contributed to the development of further ulceration and Charcot's neuropathy.

This study alluded to the fact that when a person is significantly overweight, though they are relatively inactive, the pressures going through the foot can be high but only for a short period of time. However, when a patient losses weight, their activity levels can increase and the pressures going through the foot can be less but more frequent. Combine this with an underlying osteopenia (which is often associated with a patient with overweight or obesity) [7] and the foot is more susceptible to micro trauma. Neuropathy and increased physical activity in the patient who has lost weight, combined with a decrease in bone density may increase the risk of either major or repeated microtrauma to the foot. Trauma is one of the identified risk factors for the development of acute Charcot's neuroarthropathy [7].

This case study does have limitations in that it only relates to one patient. The sample size is not generalisable and therefore there is a high risk of bias in what is being represented. The internal and external validity of the article are both poor and a high level of subjectivity is present. This article points to further research being required on the topic, with a greater sample size and more generalisable and representative results needed.

### 3.1 Post operative diabetes foot concerns for the patient with diabetes

In an article by Murchison et al. [12] the development of a Charcot's foot after significant weight reduction in people with diabetes mellitus was discussed in three case studies. The authors demonstrate that there is evidence to show that weight reduction increases strength, balance, gait and gait speed as well as changes the pressure within the foot. It is not yet known whether existing foot disease improves with substantial weight reduction. In this case study, the foot pathologies of the patients deteriorated despite significant weight reduction and improved glycaemic control after bariatric surgery. Murchison et al. [12] pointed to the risk of developing detrimental effects of chronic hyperglycaemia such as microvascular complications, notwithstanding the achievement of good glycaemic control post weight reduction. The article did conclude that diabetic neuropathy is essentially an irreversible process and although bariatric surgery may cease the progression of diabetic neuropathy, it has not been shown in the literature to substantially improve the condition. The author concluded that the combination of mineral loss and the increased mobility patients experienced due to their weight reduction, contributed to their new foot pathologies and resulting amputations. This is a point of consideration as bariatric surgery becomes more readily available to patients living with diabetes mellitus.

### 3.2 Systemic postoperative concerns of bariatric surgery

When referring to the nexus between bariatric surgery and bone mineral loss, Corbeels et al. [20] discusses the potential for even patients without diabetes to experience intestinal malabsorption as a result of bariatric surgery. In this case report, it was described that impaired intestinal absorption of calcium and vitamin D can challenge calcium homeostasis and possibly contribute to bone loss leading to an increased fracture risk. Further, hormonal changes can occur postoperatively and can also be the source of observed bone loss. This scenario would possibly result in many challenges for the patient experiencing diabetes mellitus, who would already have an increased risk of osteopenia and abnormal bony changes occurring systemically.

Despite some logical assumptions, as a stand-alone case report this article has a high level of bias. It is not generalisable or representative to the wider population as the sample size is too small and reflects only one person's experience. A broader sample size is required to make generalised statements on the effect of bariatric surgery as a weight reduction solution for the patient with overweight or obesity.

### 3.3 Postoperative foot drop

The extent to which the diabetes foot is affected by weight reduction requires further avenues for research. As an adjunct to the little that is understood, Weyns et al. [6], investigated 160 patients between January 1995 and December 2005 who were operated on for persisting foot drop. Of these 160 patients, 43.5 % (78 patients) developed their pathology after a period of serious weight reduction. The reasons for weight reduction were varied and included dieting, bariatric surgery, and severe illness. The time period for weight reduction was 2 weeks–18 months, with a mean time of 4 months. This group of 160 patients was compared with a control group of patients who underwent bariatric surgery but did not develop peroneal neuropathy. In comparison, the control group's weight reduction ranged from 10 months to 36 months, with a mean period of 21.7 months. Hence, for the control group the time for weight reduction occurred over a longer period of time. This article expresses that the relationship of weight reduction to the development of foot drop is clearly demonstrated in the population in which weight reduction occurred in a very short time frame.

Significant rapid weight reduction is associated with a higher risk of developing foot drop [6]. It is therefore recommended that slow weight reduction (which would be considered 0.5–1 kg per week) is optimal to avoid the potentiation of this disabling problem.

### 3.4 Weight reduction and plantar loading

In a randomised control trial focusing on the effects of weight reduction on the foot structure and function of patients with obesity, Song et al. [21] attempts to explain the association between obesity and foot pain caused by aberrant foot biomechanics and increased forces in patients who are obese but otherwise healthy. In this three-month randomised controlled trial, 41 participants were randomised to receive either a weight reduction intervention based on portion-controlled meals, or a delayed treatment control. The intervention group experienced significantly greater weight reduction than did the delayed control group after three months. The primary treatment group experienced a statistically significant reduction in their peak plantar pressure than the control group beneath the lateral arch and the fourth metatarsal at 3 months. This change in peak plantar pressure correlated significantly with the change in weight at the second metatarsal, the third metatarsal and the medial arch at 6 months. Modest weight reduction was shown in this study to significantly reduce the dynamic plantar loading in patients with obesity. Weight reduction, however appeared to have no effect on foot structure and gait for any of the participants to the study. The findings of this study are consistent with the findings of Hills et al. [22] who observed that BMI was correlated linearly with midfoot plantar pressures. It is of importance to note that the magnitude of weight reduction and the underlying biomechanical foot type may influence any changes in plantar loading.

Some limitations of the study however include the small sample size of 40 participants, the modest weight reduction and stringent subject criteria. This article also did not discuss the pace at which weight was lost and how it affected the foot.

### 3.5 Risks of weight reduction for the patient

The two case studies of specific relevance [12] and [7] appear to represent clinical concerns and risks to the patient with diabetes mellitus who attempts to lose weight either by diet and/or exercise or bariatric surgery. The potential for a patient with diabetes mellitus to develop neuropathy, foot deformity, macro and microvascular disease, ulceration and Charcot foot is already extremely high if hyperglycaemia is poorly controlled. Such poor outcomes can also be contributed to by the patient suffering from osteopenia and increasing their activity levels with their newfound ability to move.

For patients with diabetes mellitus, weight reduction is ultimately pursued to ameliorate health by decreasing body weight, improving health risks and ultimately improving glucose control and correcting perpetuating freely circulating high sugars within the body. With this purpose in mind, the manner in which weight reduction is undertaken is extremely important to the outcome of the patient's health (and notably their feet). It could be inferred from the articles previously mentioned that bariatric surgery can contribute to excessively fast weight reduction and malabsorption of minerals within the bones systemically. Further research is necessary to explore the potential negative outcomes of surgical weight reduction on diabetes foot complications to better understand postoperative guidance for the patient with diabetes mellitus, and it would be prudent to inform them as such prior to any surgery being undertaken.

Another cause for concern with relevance to weight reduction in the patient who has diabetes mellitus, is the change in plantar pressures and the risk of foot drop in patients who again lose weight too fast for their body to accommodate change where there may be impairment in body systems already under load. This combined with increased movement due to the change in gait of the patient with diabetes mellitus could cause further trauma to the feet at a macro and micro level.

### 3.6 Recommendations for clinical practice

• The imperative need for improved monitoring of patient's feet pre and post operatively if undergoing bariatric surgery and also during and after a period of weight reduction via diet and/or exercise.
• The need for continued and long term follow up is essential for patients with diabetes mellitus undergoing weight reduction regardless of the method in which it is achieved. Tissue viability may already be poor, and the macro and microvasculature may also be compromised. Patients with pre-existing neuropathy and peripheral arterial disease will also need to be informed of the possibility of their condition remaining unchanged due to the damage already experienced by diabetes mellitus as a metabolic disease.
• Even if glycaemic parameters normalise in patients post weight reduction, they should never be lost to follow up by the podiatrist or multidisciplinary foot care team [7]. Regular review of the feet is strongly advised.

### 3.7 Limitations of this review

This scoping review was limited by the current lack of research on the topic discussed.

## 4 Conclusion

This scoping review was undertaken to review the current literature on the topic of *the effect of weight reduction on the diabetes foot*. It is clear that gaps in the literature are evident, and it follows that recommendations for pre-emptive and long term care, and avenues for further research have been specified for patients with diabetes mellitus undergoing weight reduction.

From this scoping review and the limited research available on the topic, it could be suggested that further research is needed to see if slow weight reduction may be of benefit as opposed to rapid weight reduction, and if it will assist the patient with diabetes mellitus to have a better outcome when referring to their feet and their risk of developing or further progressing osteopenia, neuropathy, ulceration, foot deformity, Charcot foot, foot drop or even amputation of the lower limb. Where patients with a diabetes foot can participate in an increase in weight bearing and ambulation due to their increased ability to move, clinicians should afford these patients close consideration and care to ensure they do so in a balanced manner that maximises the chances of improving outcomes (i.e., improving osteopenia, sarcopenia, and potentially vascular flow) while avoiding the before mentioned complications. Further information on the use of supportive insoles postoperatively in the patient with diabetes mellitus would also be ideal to determine if they reduce the incidence of postoperative complications.

• It is recommended that further generalisable research be undertaken on the specific topic of how weight reduction effects the diabetes foot, and that the findings be used to incorporate a set of guidelines that can be implemented into the foot care plan for the patient with overweight or obesity living with diabetes mellitus.
• Information on the change in plantar pressures of the patient with diabetes mellitus is required to understand how through prevention strategies, patients can be afforded positive outcomes, whether their weight reduction is undertaken through diet and or exercise or via the use of bariatric surgery.
• It could be inferred that slow weight reduction compared to rapid weight reduction, may potentially assist the patient with diabetes mellitus to have a better outcome when referring to their feet.
• Patient's suffering from diabetes mellitus should be afforded individualised pre and post weight reduction care to their feet either by a podiatrist or multidisciplinary care team.

## Author contribution

This scoping review was compiled by Janine E. Roll Ahmed with the supervisory assistance of Professor Derek Santos and Dr John Veto.

## Declaration of artificial intelligence (AI)

No AI was used in the compilation of this paper.

## Source of funding

This review was funded solely by the corresponding author.

## Competing interest statement

I Janine E. Roll Ahmed, declare that there are no competing interests whatsoever amongst the authors of this article.

## References

[1] About Diabetes (2022). https://idf.org/aboutdiabetes/what-is-diabetes/facts-figures.html.

[2] Arksey H., O'Malley L. (2005). Scoping studies: towards a methodological framework. Int J Soc Res Methodol: Theor Pract.

[3] Wing R., Lang W., Wadden T., Safford M., Knowler W., Bertoni A., Hill J., Brancati F., Peters A., Wagenknecht L. (2011). Benefits of modest weight reduction in improving cardiovascular risk factors in overweight and obese individuals with type 2 diabetes. Diabetes Care.

[4] (2016). How does diabetes mellitus affect your feet?. Prof Nurs Today.

[5] Day C., Bailey C. (2011). Obesity in the pathogenesis of type 2 diabetes. Br J Diabetes Vasc Dis.

[6] Weyns F., Beckers F., Vanormelingen L., Vandersteen M., Niville E. (2007). Foot drop as a complication of weight reduction after bariatric surgery: is it preventable?. Obes Surg.

[7] Gooday K., Murchison R., Dhatariya K. (2014). Complex relationships requiring long term follow up: obesity, bariatric surgery-induced diabetic remission and the diabetic foot. Diabesity in Practice.

[8] Han S., Boyko E. (2018). The evidence for an obesity paradox in type 2 diabetes mellitus. Diabetes and Metabolism Journal.

[9] Piva S., Susko A., Khoja S., Josbeno D., Fitzgerald K., Toledo F. (2015). Links between osteoarthritis and diabetes: implications for management from a physical activity perspective. Clinical Geriatric Medicine.

[10] Louati K., Vidal C., Berenbaum F., Sellam J. (2015). Association between diabetes mellitus and osteoarthritis: systematic literature review and meta-analysis. Rheumatic & Musculoskeletal Diseases.

[11] Lementowski P.W., Zelicof S.B. (2008). Obesity and osteoarthritis. Am J Orthoped.

[12] Murchison R., Gooday K., Dhatariya K. (2014). The development of a Charcot foot after significant weight reduction in people with diabetes, three cautionary tales. J Am Podiatr Med Assoc.

[13] Andreoli B., Mantovani A., Andreoli C. (2020). Type 2 Diabetes, sarcopenic obesity and Mediterranean food pattern: considerations about the therapeutic effect and the problem of maintaining weight reduction and healthy habits. The outpatient experience of two clinical cases. Journal of Clinical and Translational Endocrinology: Case Reports.

[14] Mays N., Roberts E., Popay J., Fulop N., Allen P., Clarke A., Black N. (2001). Studying the organisation and delivery of health services: research methods.

[15] Pham M., Rajic A., Mcewen S. (2014). A scoping review of scoping reviews: advancing the approach and enhancing the consistency. Res Synth Methods.

[16] Centre for Reviews and Dissemination (CRD) (2008). http://www.york.ac.uk/inst/crd/systematic_reviews_book.htm.

[17] Diabetes Feet Australia (26 October 2022). https://diabetefeetaustralia.org.

[18] Bettany-Saltikov J. (2012).

[19] (3 March 2022). Critical appraisal skills programme (CASP) checklists.

[20] Corbeels K., Verlinden L., Lannoo M., Simoens C., Matthys C., Verstuyf A., Meulemans A., Carmeliet G., Van Der Schueren B. (2018). Thin bones: vitamin D and calcium handling after bariatric surgery. BoneKEy Rep.

[21] Song J., Reagan K., Tango D.N., Vander Veur S.S., Furmato J., Komaroff E., Foster G.D. (2015). Effects of weight reduction on foot structure and function in obese adults: a pilot randomised controlled trial. Gait Posture.

[22] Hills A., Hennig E., Mcdonald M., Bar-Or O. (2001). Plantar pressure differences between obese and non-obese adults: a biomechanical analysis. Int J Obes.

